# Impact of acute-phase complications and interventions on 6-month survival after stroke. A prospective observational study

**DOI:** 10.1371/journal.pone.0194786

**Published:** 2018-03-23

**Authors:** Antonio Di Carlo, Maria Lamassa, Marco Franceschini, Francesca Bovis, Lorenzo Cecconi, Sanaz Pournajaf, Stefano Paravati, Annibale Biggeri, Domenico Inzitari, Salvatore Ferro, on behalf of the Italian Study Group on Implementation of Stroke Care

**Affiliations:** 1 Institute of Neuroscience, Italian National Research Council, Florence, Italy; 2 Department of NEUROFARBA, Neuroscience Section, University of Florence, Florence, Italy; 3 IRCCS San Raffaele Pisana, Rome, Italy; 4 San Raffaele University, Rome, Italy; 5 Biostatistics Unit, Department of Health Sciences (DISSAL), University of Genoa, Genoa, Italy; 6 DISIA Department, University of Florence, Florence, Italy; 7 Department of Hospital Services, Emilia-Romagna Region Health Authority, Bologna, Italy; University of Glasgow, UNITED KINGDOM

## Abstract

The outcome of stroke patients is complex and multidimensional. We evaluated the impact of acute-phase variables, including clinical state, complications, resource use and interventions, on 6-month survival after first-ever stroke, taking into account baseline conditions exerting a possible effect on outcome.

As part of a National Research Program, we performed a prospective observational study of acute stroke patients in four Italian Regions. Consecutive patients admitted for a period of 3 months to the emergency rooms of participating hospitals were included.

A total of 1030 patients were enrolled (median age 76.0 years, 52.1% males). At 6 months, 816 (79.2%) were alive, and 164 (15.9%) deceased. Survival status at the 6-month follow-up was missing for 50 (4.9%). Neurological state in the acute phase was significantly worse in patients deceased at 6 months, who showed also higher frequency of acute-phase complications. Cox regression analysis adjusted for demographics, pre-stroke function, baseline diseases and risk factors, indicated as significant predictors of 6-month death altered consciousness (HR, 1.70; 95% CI, 1.14–2.53), total anterior circulation infarct (HR, 2.13; 95% CI, 1.44–3.15), hyperthermia (HR, 1.70; 95% CI, 1.18–2.45), pneumonia (HR, 1.76; 95% CI, 1.18–2.61), heart failure (HR, 2.87; 95% CI, 1.34–6.13) and nasogastric feeding (HR, 2.35; 95% CI, 1.53–3.60), while antiplatelet therapy during acute phase (HR, 0.56; 95% CI, 0.39–0.79), and early mobilisation (HR, 0.55; 95% CI, 0.36–0.84) significantly increased 6-month survival.

In a prospective observational study, stroke severity and some acute-phase complications, potentially modifiable, significantly increased the risk of 6-month death, independently of baseline variables. Early mobilisation positively affected survival, highlighting the role of early rehabilitation after stroke.

## Introduction

The outcome of stroke patients is complex and multidimensional, and depends on a number of pre-stroke and acute-phase variables. Baseline determinants include demographics,[1,2] vascular risk factors,[3,4] pre-existing comorbidities and functional status.[5] Considering acute-phase determinants, some are intrinsic to the stroke event itself, such as clinical and pathological types, which influence the severity of presentation.[6,7] Others depend on the interventions and procedures specifically adopted in the different settings,[8,9] as well as on the occurrence of stroke-related complications during hospitalisation. Complications during the acute phase include both medical and neurological complications. They influence length of hospital stay, access to rehabilitation, survival, functional outcome and overall costs of stroke care.[10,11]

So far, studies on stroke outcome have often evaluated the role of single acute-phase variables,[8,9,12–14] reporting on their frequency and effect on clinical course, while a more global framework of acute-phase variables with a potential impact on long-term outcome, taking also into account the role of baseline pre-stroke determinants, is lacking.

Implementation of evidence-based interventions in stroke care is receiving increasing attention, due to the perceived necessity to narrow the gap between research findings and everyday practice.[15]

The Emilia-Romagna Region, supported by the Italian Ministry of Health, coordinated the National Research Program: “Taking charge of stroke patients: implementation of integrated pathways of care and management tools”. The Program included a prospective observational study in four Italian Regions. The present research aims to evaluate the impact of acute-phase variables on survival 6 months after a first-ever stroke, taking into account baseline conditions exerting a possible impact on the selected outcome.

## Methods

### Study design

We performed a prospective observational study on stroke care in Italy in 19 hospitals located in four Regions of Northern and Central parts of the country: Emilia-Romagna, Toscana, Umbria and Lazio. All hospitals had an emergency department and a minimum of 50 acute stroke patients discharged per year; five were university hospitals, the remaining were large community hospitals having a wide range of specialties and providing advanced diagnostic procedures (e.g., magnetic resonance diagnostics).

Starting from November 1st, 2012, and for the subsequent 3 months, all consecutive patients admitted to the emergency rooms of participating hospitals with a confirmed diagnosis of first-ever stroke were included. Exclusion criteria were age <18 years, in-hospital stroke, and death within 24 hours of admission.

Stroke was defined according to World Health Organization,[16] and classified into cerebral infarction, primary intracerebral haemorrhage, and subarachnoid haemorrhage, based on at least one of the following: brain imaging (CT/MRI) performed within 30 days of stroke onset, cerebrospinal fluid analysis, or necropsy examination. Cases without subtype confirmation were unspecified stroke. Following the Oxfordshire Community Stroke Project criteria,[17] ischemic stroke subtypes were classified as total anterior circulation infarct (TACI), partial anterior circulation infarct (PACI), posterior circulation infarct (POCI) or lacunar infarct (LACI).

### Data collection

Specially trained physicians collected data in participating hospitals during the acute-phase (defined as length of stay in the ward of admission before discharge home, to rehabilitation ward, long-term care, or in-hospital death), and at the 6 months follow-up. To minimize across units and interobserver variability, a centralised training was organized to explain research design and study protocol to fieldworkers. Standardized protocols for case-ascertainment were provided, and common data collection tools were used. Problems occurring during data collection were discussed with the coordinating centers (Bologna and Florence). Data gathering was performed through structured questionnaires directly from the patient/family and hospital records. Fieldworkers had access to the dataset but were not aware of planned analyses. Information was collected on the following variables:

1. Baseline characteristics: age, sex, living conditions (home alone, with others, in institution), education, pre-stroke functional status evaluated with the modified Rankin scale,[18] and drug usage before stroke.
2. Vascular risk factors and comorbid conditions: hypertension (previous diagnosis, current treatment, or values ≥140/90 mmHg in at least 2 subsequent measurements), previous myocardial infarction, cardiac valvular disease, atrial fibrillation (medical history and/or positive ECG), heart failure, diabetes mellitus (previous diagnosis or on diabetic medication), hypercholesterolemia, smoking (current or former), peripheral artery disease, and transient ischemic attack.
3. Clinical state: neurological examination and assessment of clinical severity, evaluated with the National Institutes of Health Stroke Scale (NIHSS).[19] The NIHSS score was categorized into three classes: 0–5 (low severity), 6–13 (moderate severity), and >13 (high severity). This categorization was based on previous analyses of stroke severity in relation to outcome from population-based studies and clinical trials.[20,21]
4. Complications during acute phase: hyperglycaemia (glucose level >10 mmol/L, 180 mg/dL), hyperthermia (body temperature over 38°C), pneumonia (diagnosed by the treating physician, based on the presence of respiratory infection symptoms or signs, laboratory test and radiological evidence), pulmonary embolism, urinary retention, urinary tract infection (clinical symptoms and urine sample positive for nitrite, leucocyturia, and significant bacteriuria), heart failure (diagnosed by a cardiologist considering symptoms, signs, electrocardiography, chest X-ray, echocardiography), myocardial infarction, deep vein thrombosis (ultrasonography, with examination of the deep, superficial, and common femoral and popliteal veins), pressure ulcers, seizures.
5. Diagnostic tests (brain imaging, carotid duplex scan, transcranial Doppler, transthoracic and transoesophageal echocardiogram), resource use and treatments during hospitalisation, including early mobilisation (out-of-bed performed activities: sitting, sit-to-stand transfers, standing and walking) of patients within 48 hours of stroke onset, and length of hospital stay.

Functional status at discharge was evaluated using the Barthel Index,[22] with the following categorization: moderately or severely dependent (0–74), mildly dependent (75–84), independent ≥85.[23]

The 6-month follow-up questionnaire was administered through a face-to-face (patient still in hospital or in outpatients clinic) or telephone direct or proxy interview. The study was approved by the ethics committee of the S. Orsola-Malpighi Hospital in Bologna, the national coordinating center, on February 14th 2012, Code 47/2012/O/Oss. Patients were evaluated for altered consciousness, cognitive or language deficits. Written informed consent was given directly by patients in absence of deficits, or by legal representatives or family members according to institutional guidelines.

### Statistical analysis

Continuous variables are presented as median and 1^st^ and 3^rd^ quartiles, categorical variables as proportions. The chi-square test was used to compare categorical variables, and the Mann-Whitney U test for continuous variables. Cox proportional hazard models evaluated the independent effect of acute-phase variables on the risk of death 6 months after stroke. Hazard ratio (HR) and 95% confidence intervals (95% CI) were calculated. All variables showing statistically significant differences in univariate analysis, including demographics, pre-stroke function, baseline diseases and risk factors, were entered in the multivariate analyses, performed using a forward stepwise method (P entry = 0.05, P removal = 0.10). Intercorrelation was tested in order to avoid collinearity. All P-values were based on a 2-sided test and a significance level of < .05.

Kaplan-Meier estimates and log-rank statistics evaluated cumulative risk of death at 6 months. Survival time was measured starting from date of stroke onset to death within the follow-up period for deceased patients. Survivors were censored at their date of follow-up interview. Analyses were performed using IBM-SPSS (Statistical Package for the Social Sciences), Version 23.0 (Armonk, NY: IBM Corp.).

## Results

During the study period, 1030 patients with first-ever stroke were enrolled in participating hospitals [median age 76.0, (65.9–82.9) years; 52.1% males]. At 6 months 816 (79.2%) were alive, and 164 (15.9%) deceased. Information on survival status was missing for 50 patients (4.9%), which had complete baseline assessment, and were not significantly different from followed patients considering age [median age 79.9, (73.1–83.5) vs. 75.8 (65.6–82.9) years, P = 0.107 ], sex (males, 50.0% vs. 52.2%, P = 0.757), stroke severity (NIHSS distribution 0–5, 58.0%, 6–13, 28.0%, >13, 14.0%, vs. 51.6%, 27.2%, and 21.2%, respectively, P = 0.239). Survival was not significantly different between the 2 types of hospitals (84.1% in university hospitals vs. 82.9% in large community hospitals, P = 0.660).

Table 1 reports the frequency of baseline variables by survival status at follow-up.

**Table 1:** Distribution of baseline variables among survivors and deceased at 6 months from acute stroke.

Variable	Survivors n = 816	Deceased n = 164	P	Total sample n = 980
**Median age (1**^**st**^**-3**^**st**^ **quartiles), y**	74.4 (64.0–81.6)	82.7 (76.8–88.1)	<0.001	75.8 (65.6–82.9)
**Sex (males)**	53.2%	47.6%	0.188	52.2%
**Living at home alone**	20.2%	15.0%	0.130	19.3%
**Living at home with others**	78.9%	79.4%	0.905	79.0%
**Institutionalized**	0.9%	5.6%	<0.001	1.7%
**High school level or higher**	26.0%	12.8%	<0.001	23.8%
**Hypertension**	73.9%	87.2%	<0.001	76.1%
**Previous myocardial infarction**	11.2%	15.2%	0.139	11.8%
**Cardiac valvular disease**	6.4%	9.1%	0.199	6.8%
**Atrial fibrillation**	17.4%	31.7%	<0.001	19.8%
**Heart failure**	3.3%	8.5%	0.002	4.2%
**Diabetes**	20.6%	24.4%	0.277	21.2%
**Hypercholesterolemia**	36.4%	22.0%	<0.001	34.0%
**Current or previous smoking**	50.8%	46.2%	0.330	50.1%
**Peripheral artery disease**	5.8%	9.1%	0.104	6.3%
**Transient ischemic attack**	7.0%	7.3%	0.880	7.0%
**Antihypertensive therapy**	64.3%	79.3%	<0.001	66.8%
**Antiplatelet therapy**	36.2%	45.1%	0.031	37.7%
**Anticoagulant therapy**	7.2%	14.6%	0.002	8.5%
**Antidiabetic drugs**	16.2%	18.3%	0.506	16.5%
**Cholesterol lowering drugs**	22.5%	19.5%	0.392	22.0%
**Pre-stroke modified Rankin 3–5**	5.3%	26.3%	<0.001	8.7%

Survivors were younger, less often institutionalized prior to stroke, and with a higher education level. Among comorbidities, hypertension, atrial fibrillation and heart failure were significantly more frequent at baseline in deceased patients, as well as a higher level of prestroke functional deficits assessed by the modified Rankin scale. Prior to stroke antihypertensive, antiplatelet and anticoagulant therapy was also significantly more often reported in deceased patients.

Median length of acute stay was 9 (6–12) days in survivors and 10 (6–16) days for deceased patients (P = 0.010). Table 2 indicates that acute-phase clinical state was significantly more severe in patients deceased at 6 months.

**Table 2:** Clinical state at time of maximum impairment among survivors and deceased at 6 months from acute stroke.

Variable	Survivors n = 816	Deceased n = 164	P	Total sample n = 980
**Altered consciousness**	9.8%	46.7%	<0.001	15.7%
**Motor deficits**	66.8%	85.5%	<0.001	69.7%
**Visual field deficits**	19.4%	42.2%	<0.001	23.0%
**Aphasia**	30.8%	59.2%	<0.001	35.3%
**Dysarthria**	31.0%	24.5%	0.120	30.0%
**Dysphagia**	23.2%	62.2%	<0.001	29.7%
**Urinary incontinence**	8.6%	22.0%	<0.001	10.8%
***NIH Stroke Scale***			<0.001*	—
**0–5**	57.7%	19.7%		51.6%
**6–13**	28.2%	21.7%		27.2%
**>13**	14.1%	58.6%		21.2%
***Stroke pathological type***				
**Ischemic stroke**	84.9%	69.5%	<0.001	82.3%
**Intracerebral haemorrhage**	12.8%	26.8%	<0.001	15.1%
**Subarachnoid haemorrhage**	1.7%	2.5%	0.529	1.9%
**Unspecified stroke**	0.6%	1.2%	0.400	0.7%
***Syndromes of ischemic stroke***				
**TACI**	7.5%	31.1%	<0.001	11.4%
**PACI**	39.6%	18.9%	<0.001	36.1%
**POCI**	15.3%	9.1%	0.039	14.3%
**LACI**	16.0%	4.3%	<0.001	14.1%
**Unspecified ischemic stroke**	6.5%	6.1%	0.850	6.4%

* P for trend

They showed significantly more often altered consciousness, motor and visual field deficits, aphasia, dysphagia and urinary incontinence. NIHSS score was also significantly higher in deceased patients. Intracerebral haemorrhage was significantly more frequent in deceased patients, as well as a diagnosis of TACI among the clinical syndromes of ischemic stroke.

Complications in the acute phase are reported in Table 3.

**Table 3:** Complications in acute phase among survivors and deceased at 6 months from acute stroke.

Variable	Survivors n = 816	Deceased n = 164	P	Total sample n = 980
**Hyperglycaemia**	3.3%	9.1%	0.001	4.3%
**Hyperthermia**	10.4%	39.6%	<0.001	15.3%
**Pneumonia**	5.3%	24.4%	<0.001	8.5%
**Pulmonary embolism**	0.4%	0.6%	0.657	0.4%
**Urinary retention**	2.3%	6.1%	0.009	3.0%
**Urinary tract infections**	7.7%	17.1%	<0.001	9.3%
**Heart failure**	1.0%	6.7%	<0.001	1.9%
**Myocardial infarction**	0.2%	1.2%	0.074	0.4%
**Deep vein thrombosis**	3.2%	1.8%	0.349	3.0%
**Pressure ulcers**	2.9%	9.8%	<0.001	4.1%
**Seizures**	1.8%	7.9%	<0.001	2.9%

Those occurring with significantly higher frequency in deceased patients were hyperglycaemia, hyperthermia, pneumonia, urinary retention, urinary tract infections, heart failure, pressure ulcers and seizures.

Table 4 reports resource use and interventions in acute phase.

**Table 4:** Resource use and interventions during hospitalisation among survivors and deceased at 6 months from acute stroke.

Variable	Survivors n = 816	Deceased n = 164	P	Total sample n = 980
**Stroke unit admission**	60.0%	59.8%	0.944	60.0%
**Modified diet**	19.1%	17.1%	0.541	18.8%
**Nasogastric feeding**	8.5%	51.2%	<0.001	15.6%
**Percutaneous endoscopic gastrostomy**	0.9%	0.6%	0.747	0.8%
**Urinary catheterization**	27.9%	65.2%	<0.001	34.2%
**Deep vein thrombosis prophylaxis**	33.5%	43.9%	0.011	35.2%
**CT scan**	91.3%	94.5%	0.170	91.8%
**MRI**	40.2%	13.4%	<0.001	35.7%
**Carotid duplex scan**	68.4%	47.6%	<0.001	64.9%
**Transcranial Doppler**	24.0%	7.9%	<0.001	21.3%
**Transthoracic echocardiogram**	44.4%	26.8%	<0.001	41.4%
**Transoesophageal echocardiogram**	8.6%	3.0%	0.015	7.7%
**Antiplatelet therapy**	67.4%	46.3%	<0.001	63.9%
**Oral anticoagulant therapy**	12.7%	10.4%	0.398	12.3%
**Cholesterol lowering drugs**	37.6%	15.2%	<0.001	33.9%
**Insulin**	7.1%	11.6%	0.052	7.9%
**Intravenous thrombolysis**	15.1%	4.9%	<0.001	13.4%
**Intra-arterial thrombolysis**	2.5%	0.0%	0.043	2.0%
**Carotid endarterectomy**	2.2%	0.6%	0.176	1.9%
**Carotid angioplasty and stenting**	1.7%	1.8%	0.919	1.7%
**Early mobilisation**	51.1%	20.1%	<0.001	45.9%

Significantly more frequent in patients deceased at 6 months were nasogastric feeding and urinary catheterization, while significantly more frequently used in survivors resulted MRI, carotid and transcranial Doppler, transthoracic and transoesophageal echocardiograms. Antiplatelets and cholesterol lowering drugs were also more significantly often used in survivors, as well as intravenous and intra-arterial thrombolysis. Early mobilisation was reported in 51.1% of survivors and in 20.1% of patients deceased at 6 months (P<0.001).

The role of NIHSS on functional status and survival at discharge was also evaluated. In survivors, 26.2% of patients scoring 0–5 at NIHSS were in the Barthel Index group scoring 0–74, 4.8% in the group scoring 75–84, and 69.0% in those scoring ≥85. Of patients in the NIHSS category 6–13, 68.6% were in the Barthel Index group scoring 0–74, 6.2% in the group scoring 75–84, and 25.2% in those scoring ≥85. Considering patients in the NIHSS >13 category, 88.5% were in the Barthel Index group scoring 0–74, 2.7% in the group scoring 75–84, and 8.8% in those scoring ≥85 (P for trend <0.001).

In patients deceased at 6 months, but alive at discharge, 68.2% of patients scoring 0–5 at NIHSS were in the Barthel Index group scoring 0–74, 9.1% in the group scoring 75–84, and 22.7% in those scoring ≥85. All patients in NIHSS categories 6–13 and >13 were in the Barthel Index group scoring 0–74 (P for trend <0.001).

Considering patients scoring 0–5 at NIHSS, 1.6% deceased during the acute phase, compared with 3.9% of patients scoring 6–13, and 19.8% of those scoring >13 (P for trend <0.001).

Cox regression analysis evaluating the independent effect of acute-phase variables on survival, adjusted for demographics, pre-stroke function, baseline diseases and risk factors, and including indicators of stroke severity such as NIHSS and stroke subtypes, indicated that significant predictors of 6 months death were altered consciousness, TACI, hyperthermia, pneumonia, heart failure and nasogastric feeding, while performing MRI, antiplatelet therapy and early mobilisation in acute phase significantly increased the probability of survival at follow-up (Table 5).

**Table 5:** Acute-phase variables as predictors of 6-month mortality after stroke. Cox regression analysis adjusted for demographics, pre-stroke function, baseline diseases and risk factors. Total sample.

Variable	P	HR (95% CI)
**Altered consciousness**	0.009	1.70 (1.14–2.53)
**TACI**	<0.001	2.13 (1.44–3.15)
**Hyperthermia**	0.005	1.70 (1.18–2.45)
**Pneumonia**	0.005	1.76 (1.18–2.61)
**Heart failure**	0.006	2.87 (1.34–6.13)
**Nasogastric feeding**	<0.001	2.35 (1.53–3.60)
**MRI**	0.018	0.55 (0.34–0.90)
**Antiplatelet therapy**	0.001	0.56 (0.39–0.79)
**Early mobilisation**	0.005	0.55 (0.36–0.84)

To control for a possible “indication/reversal causality” effect, i.e. increased probability of patients with minor events to receive antiplatelet therapy and early mobilisation in acute phase, we performed a subgroup analysis for the three NIHSS classes of severity. The role of antiplatelets was confirmed for patients in the NIHSS group 0–5, (HR, 0.37, 95% CI, 0.17–0.80; P = 0.012), and the positive effect of early mobilisation for patients in the NIHSS group 6–13 (HR, 0.41, 95% CI, 0.18–0.94; P = 0.034), and >13 (HR, 0.46, 95% CI, 0.22–0.98; P = 0.045). More detailed information is provided in S1 Table.

We also evaluated possible predictors of ischemic or haemorrhagic stroke in two models of Cox regression analysis. For ischemic stroke, significant predictors of 6-month survival were essentially coincident with those found for total sample, as shown in Table 6.

**Table 6:** Acute-phase variables as predictors of 6-month mortality after ischemic stroke. Cox regression analysis adjusted for demographics, pre-stroke function, baseline diseases and risk factors.

Variable	P	HR (95% CI)
**Altered consciousness**	0.004	1.99 (1.25–3.17)
**Hyperthermia**	0.001	2.08 (1.34–3.25)
**Pneumonia**	0.001	2.16 (1.37–3.40)
**Nasogastric feeding**	<0.001	4.16 (2.58–6.71)
**MRI**	0.018	0.53 (0.31–0.90)
**Antiplatelet therapy**	0.001	0.49 (0.32–0.75)
**Early mobilisation**	0.019	0.55 (0.33–0.91)

Stroke severity, as evaluated with NIHSS, was a major predictor of outcome in haemorrhagic stroke, together with heart failure and modified diet (Table 7).

**Table 7:** Acute-phase variables as predictors of 6-month mortality after intracerebral haemorrhage. Cox regression analysis adjusted for demographics, pre-stroke function, baseline diseases and risk factors.

Variable	P	HR (95% CI)
**NIHSS**	0.001	
**NIHSS 6–13 (ref. 0–5)**	0.148	2.43 (0.73–8.08)
**NIHSS >13 (ref. 0–5)**	<0.001	5.35 (2.21–12.93)
**Heart failure**	0.011	7.48 (1.60–34.94)
**Modified diet**	0.007	0.17 (0.05–0.61)

**Fig 1:**
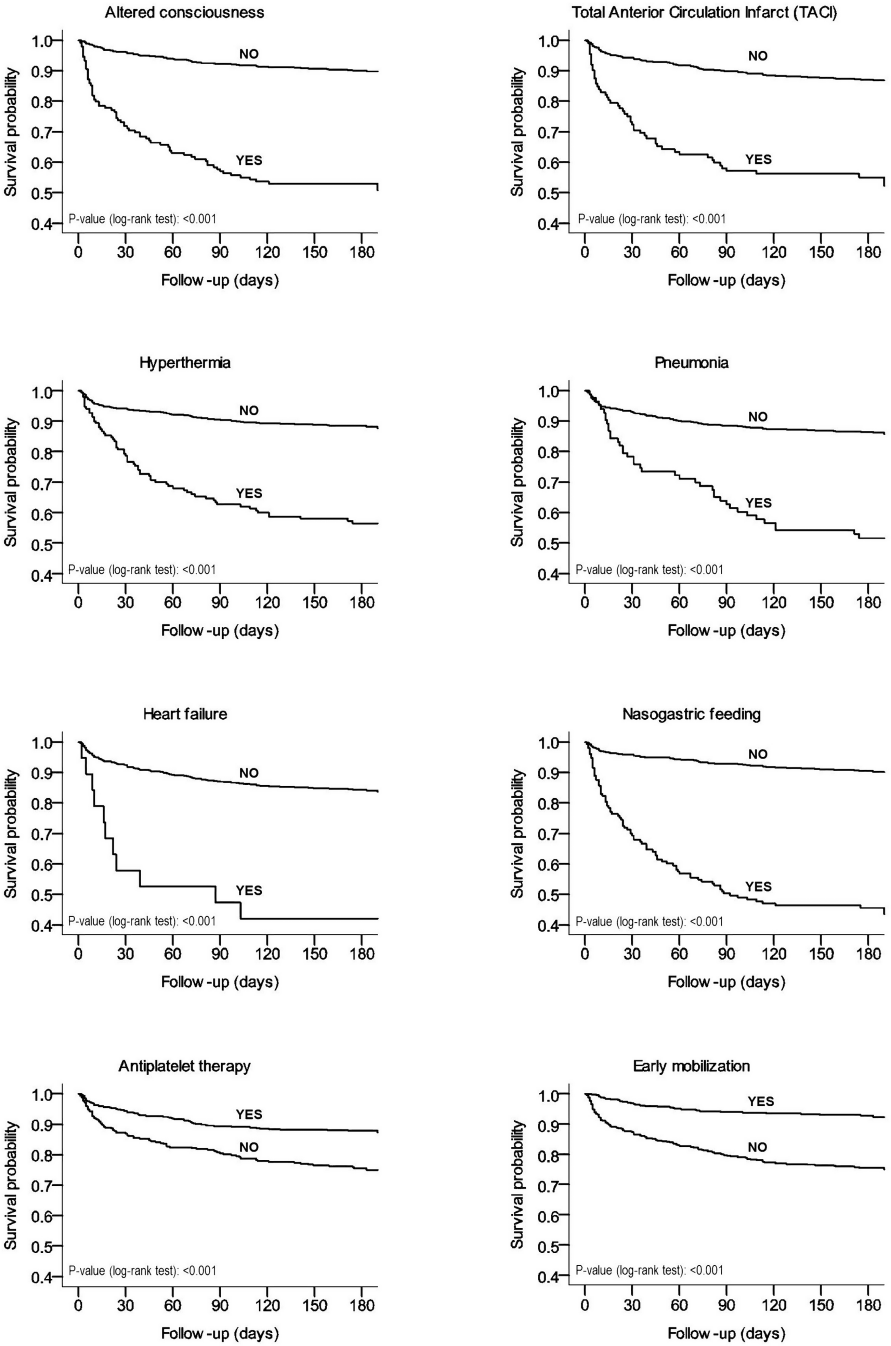
Kaplan-Meier survival estimates within 6 months after stroke onset according to acute-phase variables predictive of outcome (total sample).

Fig 1 shows the Kaplan-Meier survival curves for patients with or without acute-phase variables predictive of outcome (all log-rank tests P<0.001).

## Discussion

In a prospective survey of 980 patients hospitalised for first-ever stroke, we evaluated the role of acute-phase variables on survival 6 months after the acute event. Controlling for baseline variables with a possible influence on outcome, we found that stroke severity and acute-phase complications of hyperthermia, pneumonia, heart failure and necessity of nasogastric feeding all increased the risk of death 6 months after stroke. Improved survival was instead associated with some acute-phase interventions: performing MRI, use of antiplatelets during hospitalisation, and early mobilisation of patients.

Sixty percent of our patients were admitted to a stroke unit. In Italy, stroke patients are mainly admitted to general medicine wards, neurological wards, stroke units and geriatric wards. On a national basis, only about one third of total stroke patients are currently admitted to stroke units. The percentage, although still unsatisfactory, is higher in Northern and Central Italy, where are located more than 85% of Italian stroke units.[15] The outcome of patients admitted to stroke units was not significantly different considering patients admitted to other medical wards. However, a stroke unit was present in all but four of the hospitals included in our survey. Therefore, a treatment contamination effect may not be excluded when comparing different wards of the same hospital, with a potential dissemination of stroke units methods to the other medical wards.

Altered consciousness in acute phase reflects the severity of the stroke event, and is among the most recognized predictors of death.[24] The diagnosis of TACI is also related to a higher severity of stroke. Larger infarcts are more prone to edema development and hemorrhagic transformation,[25] and have also been associated with the presence atrial fibrillation,[6] another indicator of a worse prognosis.[3] A shorter survival in patients with TACI was reported in several surveys on stroke outcome.[6,24] Our study confirmed also the well-known role of NIHSS in predicting acute-phase and long-term mortality, particularly in patients with intracerebral haemorrhage.[26,27]

In the first days of stroke, hyperthermia occurs in up to 30–40% of patients, and it is independently associated with poor outcome and increased mortality.[28] In our patients, multivariate analysis showed that hyperthermia during acute phase increases the risk of death by 70%. Hyperthermia eight hours after stroke onset was related to a poor outcome at 3 months in the Virtual International Stroke Trials Archive (VISTA),[29] with a similar increase in the risk (HR, 1.7; 95% CI, 1.2–2.2), and it increased mortality by 50% in a meta-analysis including 14 431 patients with stroke and other brain injuries.[30]

Pneumonia is widely recognized among most frequent medical complications after stroke, with incidence ranging between 1% and 44%,[31] depending on clinical settings and heterogeneity of surveys. Stroke-associated pneumonia is thought to be secondary to aspiration and immunological alterations, part of the stroke-induced immunodepression.[32]

Although in our sample we had a low frequency of pneumonia (8.5%), this complication occurred 5 times more frequently in patients deceased at 6 months, and increased the risk of mortality at follow-up by 76% after multiple adjustments. In other surveys, stroke patients with pneumonia showed significantly higher mortality than those without after 3 months,[33] and one year from the acute event.[34]

Dysphagia is a frequent complication after stroke, reported in percentages ranging between 37% and 78% according to different surveys, probably reflecting wide differences in case-mix, study design and ascertainment.[35] Stroke patients with dysphagia are at increased risk of undernutrition and dehydration.[10] Dysphagia also significantly increases the risk of pneumonia,[35] disability, death and institutional care.[36] Modified diet and nasogastric feeding are used to meet nutritional requirements of dysphagic stroke patients: respective frequencies in our deceased patients were 17.1% and 51.2%. Only nasogastric feeding was selected in total sample and ischemic stroke as significant predictor of outcome in multivariate Cox regression, indicating that the effect on survival was probably explained by a different degree in the severity of dysphagia, while a beneficial effect of the modified diet was found in patients with intracerebral haemorrhage.

In the first months from stroke, 2% to 6.2% of patients die from cardiac causes,[37] which include acute myocardial infarction, heart failure, ventricular tachycardia/fibrillation, and cardiac arrest.[38] Despite a potentially high impact on outcome, the role of heart failure has been specifically evaluated only by few studies. In the VISTA dataset, heart failure, diabetes, high baseline creatinine, severe stroke and a long QTc or ventricular extrasystoles on ECG were predictors of severe cardiac events in the acute phase of stroke.[38] In a series of 813 consecutive stroke patients, acute-phase heart failure was reported in 5%. At 3 months after stroke, 62.5% of patients with acute heart failure were dead, vs. 16.5% of patients without heart failure.[39] In our study, acute-phase heart failure occurred in 1.9% of patients. At follow-up, 57.9% of patients with acute heart failure were dead, vs. 15.9% of stroke patients without (P<0.001). The effect of heart failure on survival was shown in total sample and in patients with a diagnosis of intracerebral haemorrhage. These data suggest the need for a careful evaluation, monitoring and management of cardiac complications after stroke.

In a recent survey of 6 regional or national stroke quality registers in Europe, brain and vascular imaging are among performance measures selected as indicators of quality of care, and possibly effective in influencing outcome.[40] However, we are very cautious about the prognostic value of MRI on outcome. Although studies showed higher sensitivity of MRI in detecting acute stroke,[41] in routine practice MRI is often used for detecting lesions in suspected stroke when CT scan results are inconclusive, as is the case for small ischemic lesions, which carry a more favourable outcome.[42] In our study MRI was performed in 20.5% of TACI and in 38.4% of LACI patients (P = 0.002). Moreover, median NIHSS was 4 (2–7) in patients with MRI performed and 7 (3–14) in patients without MRI (P<0.001).

The role of early started antiplatelet therapy in improving survival and functional outcome of ischemic stroke patients, by reducing the volume of ischemic lesion and preventing early recurrent ischemic stroke, is well established.[9] In our survey this effect was evidenced in total sample, in ischemic stroke and in less severe stroke as evaluated with NIHSS.

Early mobilisation after stroke is recommended by many guidelines, although they do not specify how soon after onset or how much therapy is best, largely due to insufficient evidence.[43] Advantages of early mobilisation include reduction of medical complications,[44] while a decrease of cerebral blood flow in critically hypoperfused regions with altered vasoregulation might explain the potentially harmful effects.[45]

While phase II results of A Very Early Rehabilitation Trial (AVERT) showed benefits of very early and intensive mobilisation after stroke on functional outcome at 3 months,[46] final results of the trial showed that the higher dose and very early mobilisation protocol was associated with reduced odds of a favourable functional outcome at 3 months.[47] In the AVERT, 92% of patients were mobilised within 24 hours in the very early mobilisation group, compared with 59% of the usual care group. In our survey, percentages were more close to the usual care group, with 46% of patients receiving early mobilisation in a longer time period of 48 hours from stroke onset. Our study adds information to the still limited evidence of early mobilisation after stroke, showing a significantly positive effect on 6-month survival after controlling for baseline and acute-phase variables. This effect was confirmed in subgroup analyses for stroke severity defined according to NIHSS score in 6–13 and >13 classes of severity.

A number of prognostic scales are available in stroke medicine, focused mainly on mortality or disability after the acute event.[48] The IScore,[49] the Acute Stroke Registry and Analysis of Lausanne (ASTRAL) Score,[50] the modified-Stroke subtype, Oxfordshire Community Stroke Project classification, Age, and prestroke modified Rankin (mSOAR),[51] are among the tools with the higher prognostic accuracy.

The IScore includes age, sex, stroke severity, stroke subtype, smoking status, preadmission dependency, and the presence or absence of atrial fibrillation, heart failure, previous myocardial infarction, cancer, renal failure on dialysis, and hyperglycemia on admission. The ASTRAL Score includes as predictors of outcome age, severity of stroke measured with the NIHSS, stroke onset to admission time, range of visual field defect, acute glucose and level of consciousness. Variables included to calculate mSOAR were age, stroke subtype (based on clinical and neuroimaging finding), Oxfordshire Community Stroke Project classification, prestroke modified Rankin Scale, and baseline NIHSS at the time of first assessment on hospital arrival.

The majority of these variables were assessed in our multivariate analyses, and some were included in our final models, although we found a prognostic role for some other acute-phase complications and interventions, such as pneumonia, hyperthermia, acute-phase heart failure and early mobilisation. Our predictors of stroke outcome are easily collected in common clinical practice, considering that the routine use of prognostic scales is often limited by their complexity or requirement of information not always available in the acute setting.[48] Accurate prediction of stroke outcome may guide physicians in treatment decision, and provide reliable information when counseling patients and their families.

Our survey has strengths and limitations. Among the strengths there is the prospective design, the short duration of enrolment, without changes in existing routine clinical practice, the large sample size and the multicentre approach. Data were collected during hospital admission by a small group of trained health professionals. To increase the homogeneity of data recording, a manual was provided to each research unit, and meetings were organized to standardize procedures. Information on survival at the 6-month follow-up was available for over 95% of patients. Studies on early complications of stroke were often focused on acute phase, while data on their impact on long-term outcome are scarce. Moreover, our study evaluated the role of multiple acute-phase variables, controlling also for baseline determinants, including demographics, vascular risk factors, pre-existing comorbidities and functional status. Among limitations there is the lack of inclusion of other acute-phase variables with a possible effect on outcome, and the possible variation of case-mix among hospitals, although we believe that, by including general and university hospitals, our sample may be considered representative of current stroke care in Northern and Central Italy.

In conclusion, in a prospective survey of patients hospitalised for first-ever stroke, controlling for baseline variables possibly influencing outcome, we found that stroke severity and some acute-phase complications, potentially modifiable, although not completely avoidable, significantly increased the risk of death at 6 months. Among acute-phase interventions, early mobilisation showed a positive effect on survival, adding information to the still limited evidence on the role of very early rehabilitation after stroke.

## Supporting information

S1 Table Antiplatelet therapy and early mobilisation in acute phase as predictors of 6-month mortality after stroke by NIHSS class of severity. Cox regression analysis adjusted for demographics, pre-stroke function, baseline diseases and risk factors, and other acute-phase variables. Total sample.
(DOCX)

## References

[1] Di Carlo A, Lamassa M, Pracucci G, Basile AM, Trefoloni G, Vanni P, et al Stroke in the very old: clinical presentation and determinants of 3-month functional outcome: A European perspective. European BIOMED Study of Stroke Care Group. Stroke. 1999;30: 2313–2319. 10548664 10.1161/01.str.30.11.2313

[2] Di Carlo A, Lamassa M, Baldereschi M, Pracucci G, Basile AM, Wolfe CD, et al; European BIOMED Study of Stroke Care Group. Sex differences in the clinical presentation, resource use, and 3-month outcome of acute stroke in Europe: data from a multicenter multinational hospital-based registry. Stroke. 2003;34: 1114–1119. doi: 10.1161/01.STR.0000068410.07397.D7 12690218 10.1161/01.STR.0000068410.07397.D7

[3] Lamassa M, Di Carlo A, Pracucci G, Basile AM, Trefoloni G, Vanni P, et al Characteristics, outcome, and care of stroke associated with atrial fibrillation in Europe: data from a multicenter multinational hospital-based registry (The European Community Stroke Project). Stroke. 2001;32: 392–398. 11157172 10.1161/01.str.32.2.392

[4] Megherbi SE, Milan C, Minier D, Couvreur G, Osseby GV, Tilling K, et al; European BIOMED Study of Stroke Care Group. Association between diabetes and stroke subtype on survival and functional outcome 3 months after stroke: data from the European BIOMED Stroke Project. Stroke. 2003;34: 688–694. doi: 10.1161/01.STR.0000057975.15221.40 12624292 10.1161/01.STR.0000057975.15221.40

[5] Koennecke HC, Belz W, Berfelde D, Endres M, Fitzek S, Hamilton F, et al; Berlin Stroke Register Investigators. Factors influencing in-hospital mortality and morbidity in patients treated on a stroke unit. Neurology. 2011;77: 965–972. doi: 10.1212/WNL.0b013e31822dc795 21865573 10.1212/WNL.0b013e31822dc795

[6] Di Carlo A, Lamassa M, Baldereschi M, Pracucci G, Consoli D, Wolfe CD, et al; European BIOMED Study of Stroke Care Group. Risk factors and outcome of subtypes of ischemic stroke. Data from a multicenter multinational hospital-based registry. The European Community Stroke Project. J Neurol Sci. 2006;244: 143–150. doi: 10.1016/j.jns.2006.01.016 16530226 10.1016/j.jns.2006.01.016

[7] Vibo R, Kõrv J, Roose M. One-year outcome after first-ever stroke according to stroke subtype, severity, risk factors and pre-stroke treatment. A population-based study from Tartu, Estonia. Eur J Neurol. 2007;14: 435–439. doi: 10.1111/j.1468-1331.2007.01704.x 17388994 10.1111/j.1468-1331.2007.01704.x

[8] Geeganage C, Beavan J, Ellender S, Bath PM. Interventions for dysphagia and nutritional support in acute and subacute stroke. Cochrane Database Syst Rev. 2012;10:CD000323 doi: 10.1002/14651858.CD000323.pub2 23076886 10.1002/14651858.CD000323.pub2

[9] Sandercock PA, Counsell C, Tseng MC, Cecconi E. Oral antiplatelet therapy for acute ischaemic stroke. Cochrane Database Syst Rev. 2014;3:CD000029. 10.1002/14651858.CD000029.pub3 PMC6669270 24668137

[10] Kumar S, Selim MH, Caplan LR. Medical complications after stroke. Lancet Neurol. 2010;9: 105–118. doi: 10.1016/S1474-4422(09)70266-2 20083041 10.1016/S1474-4422(09)70266-2

[11] Balami JS, Chen RL, Grunwald IQ, Buchan AM. Neurological complications of acute ischaemic stroke. Lancet Neurol. 2011;10: 357–371. doi: 10.1016/S1474-4422(10)70313-6 21247806 10.1016/S1474-4422(10)70313-6

[12] Emsley HC, Hopkins SJ. Acute ischaemic stroke and infection: recent and emerging concepts. Lancet Neurol. 2008;7: 341–353. doi: 10.1016/S1474-4422(08)70061-9 18339349 10.1016/S1474-4422(08)70061-9

[13] Ifejika-Jones NL, Peng H, Noser EA, Francisco GE, Grotta JC. Hospital-acquired symptomatic urinary tract infection in patients admitted to an academic stroke center affects discharge disposition. PM&R. 2013;5: 9–15. 23103046 10.1016/j.pmrj.2012.08.002

[14] Burkot J, Kopec G, Pera J, Slowik A, Dziedzic T. Decompensated heart failure is a strong independent predictor of functional outcome after ischemic stroke. J Card Fail. 2015;21: 642–646. doi: 10.1016/j.cardfail.2015.03.008 25800549 10.1016/j.cardfail.2015.03.008

[15] Di Carlo A, Pezzella FR, Fraser A, Bovis F, Baeza J, McKevitt C, et al; European Implementation Score Collaboration Study Group. Methods of implementation of evidence-based stroke care in Europe: European Implementation Score Collaboration. Stroke. 2015;46: 2252–2259. doi: 10.1161/STROKEAHA.115.009299 26111887 10.1161/STROKEAHA.115.009299

[16] Hatano S. Experience from a multicentre stroke register: a preliminary report. Bull WHO. 1976;54: 541–553. 1088404 PMC2366492

[17] Bamford J, Sandercock P, Dennis M, Burn J, Warlow C. Classification and natural history of clinically identifiable subtypes of cerebral infarction. Lancet. 1991;337: 1521–1526. 1675378 10.1016/0140-6736(91)93206-o

[18] van Swieten JC, Koudstaal PJ, Visser MC, Schouten HJ, van Gijn J. Interobserver agreement for the assessment of handicap in stroke patients. Stroke. 1988;19: 604–607. 3363593 10.1161/01.str.19.5.604

[19] Brott T, Adams HP Jr, Olinger CP, Marler GR, Barsan WG, Biller J, et al Measurements of acute cerebral infarction: a clinical examination scale. Stroke. 1989;20: 864–870. 2749846 10.1161/01.str.20.7.864

[20] Elkind MS, Cheng J, Rundek T, Boden-Albala B, Sacco RL. Leukocyte count predicts outcome after ischemic stroke: the Northern Manhattan Stroke Study. J Stroke Cerebrovasc Dis. 2004;13: 220–227. doi: 10.1016/j.jstrokecerebrovasdis.2004.07.004 17903979 10.1016/j.jstrokecerebrovasdis.2004.07.004

[21] Dhamoon MS, Sciacca RR, Rundek T, Sacco RL, Elkind MS. Recurrent stroke and cardiac risks after first ischemic stroke: the Northern Manhattan Study. Neurology. 2006;66: 641–646. doi: 10.1212/01.wnl.0000201253.93811.f6 16534100 10.1212/01.wnl.0000201253.93811.f6

[22] Mahoney FI, Barthel DW. Functional evaluation: The Barthel Index. Md State Med J. 1965;14: 61–65. 14258950

[23] Di Carlo A, Inzitari D, Galati F, Baldereschi M, Giunta V, Grillo G, et al A prospective community-based study of stroke in Southern Italy: the Vibo Valentia incidence of stroke study (VISS). Methodology, incidence and case fatality at 28 days, 3 and 12 months. Cerebrovasc Dis. 2003;16: 410–417. doi: 10.1159/000072565 13130183 10.1159/000072565

[24] Koton S, Tanne D, Green MS, Bornstein NM. Mortality and predictors of death 1 month and 3 years after first-ever ischemic stroke: data from the first national acute stroke Israeli survey (NASIS 2004). Neuroepidemiology. 2010;34: 90–96. doi: 10.1159/000264826 20016218 10.1159/000264826

[25] Sandset EC, Jusufovic M, Sandset PM, Bath PM, Berge E; SCAST Study Group. Effects of blood pressure-lowering treatment in different subtypes of acute ischemic stroke. Stroke. 2015;46: 877–879. doi: 10.1161/STROKEAHA.114.008512 25657183 10.1161/STROKEAHA.114.008512

[26] Cheung RT, Zou LY. Use of the original, modified, or new intracerebral hemorrhage score to predict mortality and morbidity after intracerebral hemorrhage. Stroke. 2003;34: 1717–1722. doi: 10.1161/01.STR.0000078657.22835.B9 12805488 10.1161/01.STR.0000078657.22835.B9

[27] Satopää J, Mustanoja S, Meretoja A, Putaala J, Kaste M, Niemelä M, et al Comparison of all 19 published prognostic scores for intracerebral hemorrhage. J Neurol Sci. 2017;379: 103–108. doi: 10.1016/j.jns.2017.05.034 28716217 10.1016/j.jns.2017.05.034

[28] Campos F, Sobrino T, Vieites-Prado A, Pérez-Mato M, Rodríguez-Yáñez M, Blanco M, et al Hyperthermia in human ischemic and hemorrhagic stroke: similar outcome, different mechanisms. PLoS One. 2013;8:e78429 doi: 10.1371/journal.pone.0078429 24223804 10.1371/journal.pone.0078429 PMC3817202

[29] Saini M, Saqqur M, Kamruzzaman A, Lees KR, Shuaib A; VISTA Investigators. Effect of hyperthermia on prognosis after acute ischemic stroke. Stroke. 2009;40: 3051–3059. doi: 10.1161/STROKEAHA.109.556134 19644066 10.1161/STROKEAHA.109.556134

[30] Greer DM, Funk SE, Reaven NL, Ouzounelli M, Uman GC. Impact of fever on outcome in patients with stroke and neurologic injury: a comprehensive meta-analysis. Stroke. 2008;39: 3029–3035. doi: 10.1161/STROKEAHA.108.521583 18723420 10.1161/STROKEAHA.108.521583

[31] Papavasileiou V, Milionis H, Smith CJ, Makaritsis K, Bray BD, Michel P, et al External Validation of the Prestroke Independence, Sex, Age, National Institutes of Health Stroke Scale (ISAN) Score for Predicting Stroke-Associated Pneumonia in the Athens Stroke Registry. J Stroke Cerebrovasc Dis. 2015;24: 2619–2624. doi: 10.1016/j.jstrokecerebrovasdis.2015.07.017 26347399 10.1016/j.jstrokecerebrovasdis.2015.07.017

[32] Hannawi Y, Hannawi B, Rao CP, Suarez JI, Bershad EM. Stroke-associated pneumonia: major advances and obstacles. Cerebrovasc Dis. 2013;35: 430–443. doi: 10.1159/000350199 23735757 10.1159/000350199

[33] Aslanyan S, Weir CJ, Diener HC, Kaste M, Lees KR; GAIN International Steering Committee and Investigators. Pneumonia and urinary tract infection after acute ischaemic stroke: a tertiary analysis of the GAIN International trial. Eur J Neurol. 2004;11: 49–53. 14692888 10.1046/j.1468-1331.2003.00749.x

[34] Hilker R, Poetter C, Findeisen N, Sobesky J, Jacobs A, Neveling M, et al Nosocomial pneumonia after acute stroke: implications for neurological intensive care medicine. Stroke. 2003;34: 975–981. doi: 10.1161/01.STR.0000063373.70993.CD 12637700 10.1161/01.STR.0000063373.70993.CD

[35] Martino R, Foley N, Bhogal S, Diamant N, Speechley M, Teasell R. Dysphagia after stroke: incidence, diagnosis, and pulmonary complications. Stroke. 2005;36: 2756–2763. doi: 10.1161/01.STR.0000190056.76543.eb 16269630 10.1161/01.STR.0000190056.76543.eb

[36] Cohen DL, Roffe C, Beavan J, Blackett B, Fairfield CA, Hamdy S, et al Post-stroke dysphagia: A review and design considerations for future trials. Int J Stroke. 2016;11:399–411. doi: 10.1177/1747493016639057 27006423 10.1177/1747493016639057

[37] Adams RJ, Chimowitz MI, Alpert JS, Awad IA, Cerqueria MD, Fayad P, et al; American Heart Association/American Stroke Association. Coronary risk evaluation in patients with transient ischemic attack and ischemic stroke: a scientific statement for healthcare professionals from the Stroke Council and the Council on Clinical Cardiology of the American Heart Association/American Stroke Association. Stroke. 2003;34: 2310–2322. doi: 10.1161/01.STR.0000090125.28466.E2 12958318 10.1161/01.STR.0000090125.28466.E2

[38] Prosser J, MacGregor L, Lees KR, Diener HC, Hacke W, Davis S; VISTA Investigators. Predictors of early cardiac morbidity and mortality after ischemic stroke. Stroke. 2007;38: 2295–2302. doi: 10.1161/STROKEAHA.106.471813 17569877 10.1161/STROKEAHA.106.471813

[39] Micheli S, Agnelli G, Caso V, Alberti A, Palmerini F, Venti M, et al Acute myocardial infarction and heart failure in acute stroke patients: frequency and influence on clinical outcome. J Neurol. 2012;259: 106–110. doi: 10.1007/s00415-011-6136-4 21691731 10.1007/s00415-011-6136-4

[40] Norrving B, Bray BD, Asplund K, Heuschmann P, Langhorne P, Rudd AG, et al Cross-National Key Performance Measures of the Quality of Acute Stroke Care in Western Europe. Stroke. 2015;46: 2891–2895. doi: 10.1161/STROKEAHA.115.008811 26265128 10.1161/STROKEAHA.115.008811

[41] Audebert HJ, Fiebach JB. Brain imaging in acute ischemic stroke. MRI or CT? Curr Neurol Neurosci Rep. 2015;15: 6. 10.1007/s11910-015-0526-4 25663034

[42] Lövblad KO, Altrichter S, Mendes Pereira V, Vargas M, Marcos Gonzalez A, Haller S, et al Imaging of acute stroke: CT and/or MRI. J Neuroradiol. 2015;42: 55–64. doi: 10.1016/j.neurad.2014.10.005 25466468 10.1016/j.neurad.2014.10.005

[43] van Wijk R, Cumming T, Churilov L, Donnan G, Bernhardt J. An early mobilization protocol successfully delivers more and earlier therapy to acute stroke patients: further results from phase II of AVERT. Neurorehabil Neural Repair. 2012;26: 20–26. doi: 10.1177/1545968311407779 21807984 10.1177/1545968311407779

[44] Diserens K, Moreira T, Hirt L, Faouzi M, Grujic J, Bieler G, et al Early mobilization out of bed after ischaemic stroke reduces severe complications but not cerebral blood flow: a randomized controlled pilot trial. Clin Rehabil. 2012;26: 451–459. doi: 10.1177/0269215511425541 22144725 10.1177/0269215511425541

[45] Olavarría VV, Arima H, Anderson CS, Brunser AM, Muñoz-Venturelli P, Heritier S, et al Head position and cerebral blood flow velocity in acute ischemic stroke: a systematic review and meta-analysis. Cerebrovasc Dis. 2014;37: 401–408. doi: 10.1159/000362533 24993471 10.1159/000362533

[46] Cumming TB, Thrift AG, Collier JM, Churilov L, Dewey HM, Donnan GA, et al Very early mobilization after stroke fast-tracks return to walking: further results from the phase II AVERT randomized controlled trial. Stroke. 2011;42: 153–158. doi: 10.1161/STROKEAHA.110.594598 21148439 10.1161/STROKEAHA.110.594598

[47] Bernhardt J, Langhorne P, Lindley RI, Thrift AG, Ellery F, Collier J, et al; AVERT Trial Collaboration group. Efficacy and safety of very early mobilisation within 24h of stroke onset (AVERT): a randomised controlled trial. Lancet. 2015;386: 46–55. doi: 10.1016/S0140-6736(15)60690-0 25892679 10.1016/S0140-6736(15)60690-0

[48] Quinn TJ, Singh S, Lees KR, Bath PM, Myint PK; VISTA Collaborators. Validating and comparing stroke prognosis scales. Neurology. 2017;89: 997–1002. doi: 10.1212/WNL.0000000000004332 28794250 10.1212/WNL.0000000000004332

[49] Saposnik G, Kapral MK, Liu Y, Hall R, O'Donnell M, Raptis S, et al IScore: a risk score to predict death early after hospitalization for an acute ischemic stroke. Circulation. 2011;123: 739–749. doi: 10.1161/CIRCULATIONAHA.110.983353 21300951 10.1161/CIRCULATIONAHA.110.983353

[50] Ntaios G, Faouzi M, Ferrari J, Lang W, Vemmos K, Michel P. An integer-based score to predict functional outcome in acute ischemic stroke: the ASTRAL score. Neurology. 2012;78: 1916–1922. doi: 10.1212/WNL.0b013e318259e221 22649218 10.1212/WNL.0b013e318259e221

[51] Abdul-Rahim AH, Quinn TJ, Alder S, Clark AB, Musgrave SD, Langhorne P et al Derivation and Validation of a Novel Prognostic Scale (Modified-Stroke Subtype, Oxfordshire Community Stroke Project Classification, Age, and Prestroke Modified Rankin) to Predict Early Mortality in Acute Stroke. Stroke. 2016;47: 74–79. doi: 10.1161/STROKEAHA.115.009898 26578661 10.1161/STROKEAHA.115.009898

