# Supplementary material for: Impact of acute-phase complications and interventions on 6-month survival after stroke. A prospective observational study
Source: PLoS One. 2018 Mar 23;13(3):e0194786. doi: 10.1371/journal.pone.0194786 (PMC5865737; doi:10.1371/journal.pone.0194786)
Supplement: S1 Table — Cox regression analysis adjusted for demographics, pre-stroke function, baseline diseases and risk factors, and other acute-phase variables. Total sample. (DOCX) [file pone.0194786.s001.docx]

**S1 Table. Antiplatelet therapy and early mobilisation in acute phase as predictors of 6-month mortality after stroke by NIHSS class of severity. Cox regression analysis adjusted for demographics, pre-stroke function, baseline diseases and risk factors, and other acute-phase variables. Total sample.**

|  | **P** | **HR (95% CI)** |
| --- | --- | --- |
| **NIHSS 0-5** |  |  |
| **Antiplatelet therapy** | 0.012 | 0.37 (0.17-0.80) |
| **Early mobilisation** | 0.895 | 1.06 (0.48-2.32) |
|  |  |  |
| **NIHSS 6-13** |  |  |
| **Antiplatelet therapy** | 0.214 | 0.63 (0.30-1.31) |
| **Early mobilisation** | 0.034 | 0.41 (0.18-0.94) |
|  |  |  |
| **NIHSS>13** |  |  |
| **Antiplatelet therapy** | 0.599 | 0.89 (0.57-1.39) |
| **Early mobilisation** | 0.045 | 0.46 (0.22-0.98) |
